# Association of genetic polymorphisms related to Johne’s disease with estimated breeding values of Holstein sires for milk ELISA test scores

**DOI:** 10.1186/s12917-020-02381-9

**Published:** 2020-05-27

**Authors:** Sanjay Mallikarjunappa, Flavio S. Schenkel, Luiz F. Brito, Nathalie Bissonnette, Filippo Miglior, Jacques Chesnais, Michael Lohuis, Kieran G. Meade, Niel A. Karrow

**Affiliations:** 1 Centre for Genetic Improvement of Livestock, Department of Animal Biosciences, University of Guelph, Guelph, ON N1G 2W1 Canada; 2 Animal and Bioscience Research Department, Teagasc, Grange, Co. Meath, Ireland; 3 Department of Animal Sciences, Purdue University, West Lafayette, IN 47907 USA; 4 Agriculture and Agri-Food Canada, Sherbrooke Research and Development Centre, Sherbrooke, QC J1M 0C8 Canada; 5 The Semex Alliance, Guelph, ON N1G 3Z2 Canada

**Keywords:** Johne’s disease, Cattle, Paratuberculosis, SNP validation

## Abstract

**Background:**

Johne’s disease (JD) is a chronic intestinal inflammatory disease caused by *Mycobacterium avium subsp. paratuberculosis* (MAP) infection in ruminants. Since there are currently no effective vaccine or treatment options available to control JD, genetic selection may be an alternative strategy to enhance JD resistance. Numerous Single Nucleotide Polymorphisms (SNPs) have been reported to be associated with MAP infection status based on published genome-wide association and candidate gene studies. The main objective of this study was to validate these SNPs that were previously identified to be associated with JD by testing their effect on Holstein bulls’ estimated breeding values (EBVs) for milk ELISA test scores, an indirect indicator of MAP infection status in cattle.

**Results:**

Three SNPs, rs41810662, rs41617133 and rs110225854, located on *Bos taurus* autosomes (BTA) 16, 23 and 26, respectively, were confirmed as significantly associated with Holstein bulls’ EBVs for milk ELISA test score (FDR < 0.01) based on General Quasi Likelihood Scoring analysis (GQLS) analysis. Single-SNP regression analysis identified four SNPs that were associated with sire EBVs (FDR < 0.05). This includes two SNPs that were common with GQLS (rs41810662 and rs41617133), with the other two SNPs being rs110494981 and rs136182707, located on BTA9 and BTA16, respectively.

**Conclusions:**

The findings of this study validate the association of SNPs with JD MAP infection status and highlight the need to further investigate the genomic regions harboring these SNPs.

## Background

Johne’s disease (JD), also known as paratuberculosis, manifests as chronic enteritis in cattle and is caused by infection with the Gram-positive bacteria *Mycobacterium avium subsp. paratuberculosis* (MAP). With its worldwide prevalence, the disease is responsible for significant economic losses to the dairy industry [1]. Albeit controversial and debatable, JD etiological agent MAP is also viewed as a pathogen with zoonotic effects. Report of isolation of MAP from intestines of human patients suffering from Crohn’s disease has raised public health concerns [2]. Factors like non-availability of an efficacious vaccine to combat MAP infection, issues associated with currently available JD diagnostic assays such as long turnaround time associated with MAP culture tests, low sensitivity of ELISA tests during early stages of JD, and absence of efficient treatment options have limited JD control around the world [3]. Heritability estimates using different phenotypes of JD have been reported and are found to range from 0.06 to 0.27 [4–7] suggesting that there is enough genetic variation to enable selection for reduced susceptibility to MAP infection [8]. Given the low-to-moderate heritability estimates of JD resistance, the difficulty to collect accurate phenotypes on a large number of animals, and the fact that that animals are tested for MAP infection at a later life stage (i.e., do not have own record at selection stage), the use of genomic information is a promising way to make genetic progress for JD resistance.

Genome-wide association (GWAS) and candidate gene studies concerning JD have identified numerous single nucleotide polymorphisms (SNPs) across the bovine genome which are significantly associated with JD status in dairy cattle [9–17]. The identification of JD associated SNPs and the heritable nature of MAP infection reflect the genetic variation in susceptibility and resistance to JD. These identified SNPs could be used in JD resistance breeding programs based on marker-enhanced selection (MES) once they are included in genotyping platforms. However, before this can occur there is a need to validate them, especially using independent cattle populations.

In a recent study by Brito et al. [18], the authors reported genetic parameters such as heritability for MAP-specific antibody response and estimated breeding values (EBVs) for Holstein cattle based on milk ELISA test records along with their correlation with other economically important trait like milk yield; and other routinely evaluated traits such as somatic cell score (SCS); reproduction traits (calving to first service, 56-d non-return rate, Number of services, cows, First service to conception, cows, Days open; longevity trait (Direct herd life); and confirmation traits (Overall feet and legs, Overall conformation). Milk ELISA is a JD diagnostic method that detects MAP-specific antibodies in animals exposed to MAP and is therefore an indirect indicator of MAP infection status in cattle [19]. Unlike direct diagnostic tests based on MAP culture tests, MAP ELISA test have a quick turnaround time and can be easily used at the herd level. As studies concerning validation of JD SNPs are lacking, sires with highly accurate EBVs for milk ELISA testing can be used to validate previously identified JD SNPs. Therefore, the main objective of this study was to validate some of the previously associated JD SNPs in literature by testing their association with sire EBVs for milk ELISA test score.

## Results

**Fig. 1:**
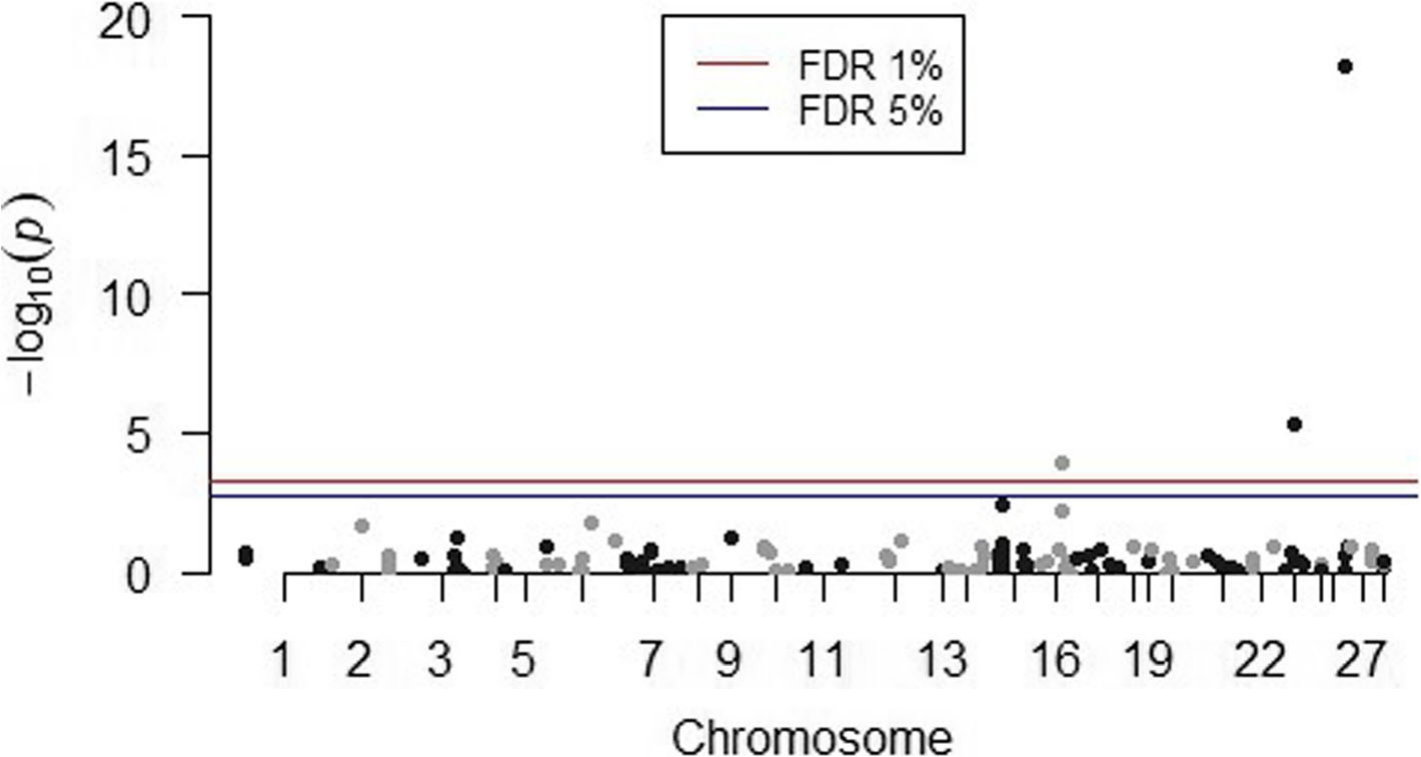
Manhattan plot for General Quasi Likelihood Score association analysis. A total of 3 SNPs across 3 chromosomes (BTA16, BTA23 and BTA26) were found to be significant at FDR < 0.01

**Fig. 2:**
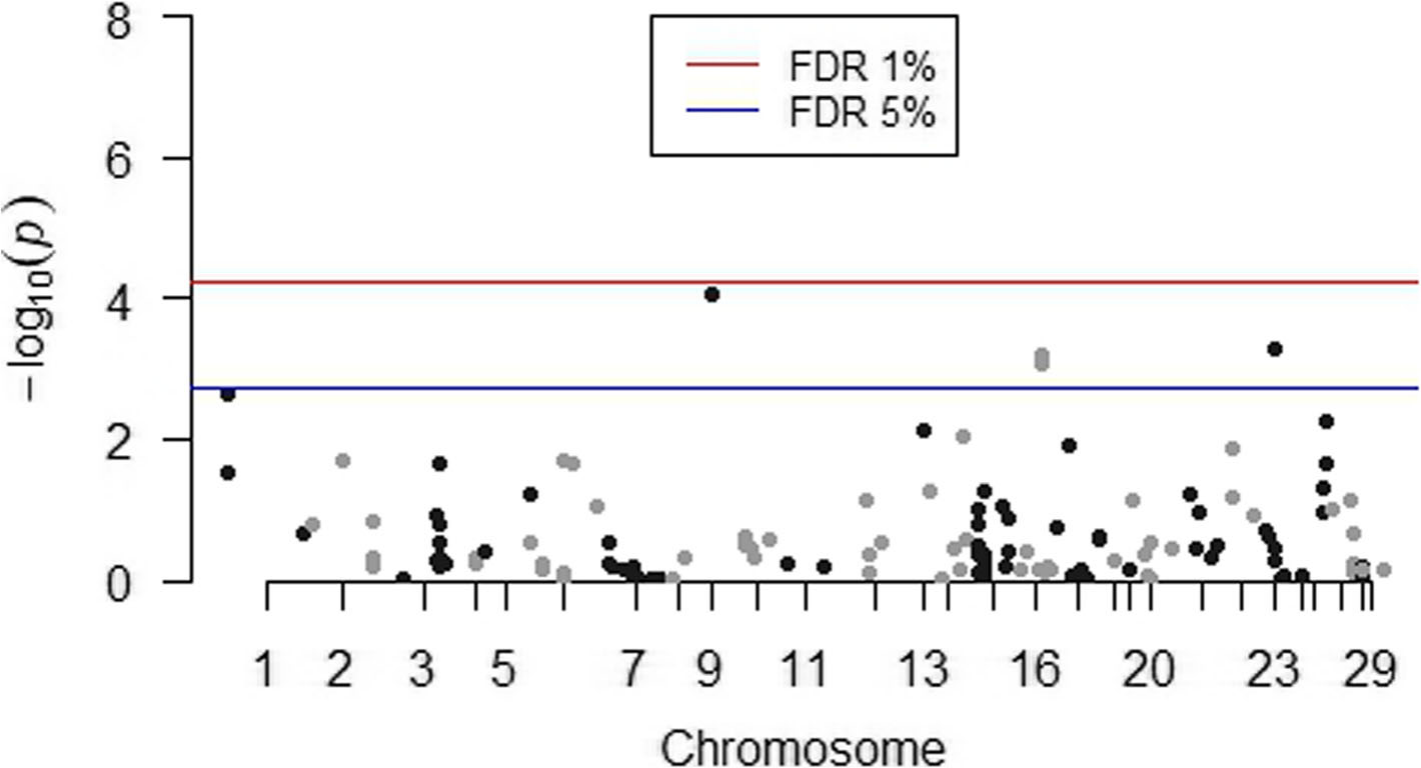
Manhattan plot for Single SNP regression analysis. A total of 4 SNPs across 3 chromosomes (BTA9, BTA16 and BTA23) were found to be significant at FDR < 0.05

A total of 141 SNPs passed the quality control test for MAF threshold and were included in the association analyses. After the General Quasi Likelihood Scoring (GQLS) association analysis, the SNPs rs41810662 (*P-value* = 0.00011), rs41617133 (*P-value* = 4.5E-06) and rs110225854 (*P-value* = 6.4E-19) were found to be significantly associated with Holstein sire EBVs for milk ELISA test scores at a FDR of 1%, and no other SNPs were found to be significant at a FDR of 5%. Table 1 lists the significant SNPs based on GQLS analysis. The Manhattan plot of GQLS analysis is shown in Fig. 1. A total of four SNPs were found to be significantly associated with sire EBVs by single-SNP regression analysis. This included the SNPs rs41810662 (*P-value* = 0.00062) and rs41617133 (*P-value* = 0.00050) that were also identified in the GQLS analysis; and two other SNPs, rs110494981 (*P-value* = 9.0E-05) and rs136182707 (*P-value* = 0.00088), which are located on BTA9 and BTA16, respectively. Table 2 lists all the significant SNPs along with their estimated SNP effect based on single-SNP regression analysis. The Manhattan plot of single SNP regression analysis is shown in Fig. 2.

**Table 1:** List of significant SNPs associated with sire EBVs for milk ELISA, based on the GQLS method

SNP rsID	SNP name	BTA	Position (bp)	MAF	*P*-value	FDR
rs41810662	BovineHD1600015492	16	55,677,310	0.30	0.00011	1%
rs41617133	Hapmap51130-BTA-105627	23	32,876,929	0.20	4.5E-06	1%
rs110225854	ARS-BFGL-NGS-114768	26	38,924,276	0.15	6.4E-19	1%

*MAF* Minor Allele Frequency; *BTA* Bos taurus autosome; *FDR* False Discovery Rate;

**Table 2:** List of significant SNPs associated with sire EBVs for milk ELISA, based on the single-SNP regression method

SNP rsID	SNP name	BTA	Position (bp)	MAF	*P*-value	FDR	b
rs110494981	ARS-BFGL-NGS-8531	9	44,713,803	0.24	9.0E-05	5%	0.00870
rs136182707	BovineHD1600014724	16	53,247,138	0.43	0.00088	5%	0.00680
rs41810662	BovineHD1600015492	16	55,677,310	0.30	0.00062	5%	0.00719
rs41617133	Hapmap51130-BTA-105627	23	32,876,929	0.20	0.00050	5%	0.00368

*MAF* Minor Allele Frequency; *BTA* Bos taurus autosome; *FDR* False Discovery Rate; b – variance explained by SNP relative to the Standard Deviation of EBVs after correcting for selective genotyping

## Discussion

With a global herd level prevalence ranging between 7 and 60% [20], Johne’s disease is a severe production limiting disease with significant animal welfare concerns to the worldwide dairy industry. Annual production losses due to JD on US dairy industry alone is estimated to be to $200–$250 millions dollars [1]. The losses associated with JD are mainly due to reduction in milk production, premature culling of JD positive animals, the management costs associated with JD control programs to limit MAP spread within and across the herds [21]. The role of host genetics in influencing JD infection status in cattle has been extensively studied, which resulted in the identification of several impactful genetic markers across the cattle genome [18]. However, studies concerning validation of genetic markers are still lacking. In this study, we validated some SNPs previously associated with JD by testing their association with breeding values estimated for JD milk ELISA test scores. A total of 498 bulls were classified into high (*n* = 248) and low (*n* = 250) groups based on their EBVs for milk ELISA test scores. Both groups were then genotyped using a customized SNP panel comprising 155 of the most prominent JD SNPs reported in the literature, including SNP from studies that used phenotypes other than milk ELISA test to define the case-control populations. Two association analyses were used to carry out SNP validation: a) General Quasi Likelihood Scoring (GQLS) analysis based on logistic regression, and, b) single-SNP regression.

Three SNPs rs41810662, rs41617133 and rs110225854 were found to be significantly associated with Holstein sire EBVs using GQLS analysis at 1% FDR. The association of these three SNPs with sire EBVs in the current study confirmed their association with JD. The SNP rs41810662 is located on BTA16 and was previously found to be associated with MAP antibody response in Holstein cows [22]. The other two SNPs (rs41617133 and rs110225854) are located on BTA23 and BTA26, respectively, and were previously reported to be associated with JD susceptibility in Holsteins [14]. These last two SNPs were selected from a GWAS study that used fecal culture along with blood ELISA to define MAP infection status. This may be indicative of the shared genomic region in Holstein cattle that influences both JD fecal culture and ELISA positivity and a stronger evidence of these SNPs’ role towards resistance to MAP infection.

A total of four SNPs were found to be significantly associated with sire EBVs by single-SNP regression analysis at 5% FDR. This included the SNPs rs41810662 and rs41617133 that were also identified in the GQLS analysis; and two other SNPs, rs110494981 and rs136182707, which are located on BTA9 and BTA16, respectively. The SNP rs110494981 was previously found to be associated with serum ELISA positivity for MAP antibodies in Italian Holsteins [13], whereas rs136182707 was previously identified by Mallikarjunappa et al. [22] and was found to be associated with MAP antibody ELISA positivity.

One main result from this study is that most of the SNPs (137 out of 141) were not found to be significant in the population under study. This is not uncommon and may have multiple causes. While several GWAS studies have enabled identification of many SNPs associated with MAP infection status in cattle, there seems to be little congruence among them, which confirms the complex polygenic nature of the disease [8, 18]. Among other factors, the choice of phenotype (e.g., milk or blood ELISA, fecal MAP culture, tissue MAP culture) used to define infection status in case-control studies is shown to impact results [23]. Each test differs in their specificity and sensitivity in accurately diagnosing MAP infection and results are often dictated by the stage of disease progression in the tested animal [24]. As a result, precise phenotyping of infected versus non-infected animals remains a challenge.

Milk ELISA is an indirect test for diagnosing MAP infection status that is based on identification of MAP-specific antibodies in MAP exposed animals. Unlike MAP culture techniques that take weeks to diagnose MAP infection, ELISA tests are more feasible at the herd level because of their rapid turn-around time and measurement cost. The objective of this study was to validate the previously reported JD SNPs reported in the literature. For this, we utilized sire EBVs estimated for milk ELISA test score to validate JD SNPs and confirm their association. As the sire EBVs used here were estimated using a large dataset of milk ELISA records, testing the association of JD SNPs with sire EBVs allows their validation at a large population level, while reducing the costs associated with genotyping large number of daughters. Genome-wide studies using EBVs to estimate SNP effects have been previously reported [25]. We adapted selective genotyping approach in our study where sires with EBVs estimated for milk ELISA results were classified into high and low groups and were subsequently genotyped. This approach of genotyping animals with extreme phenotypes reduces the need to genotype large number of animals and also increases power in determining the association of variants/SNPs in customized genotyping study like ours [26, 27].

Only few SNPs were validated while using EBVs estimated for milk ELISA test results. The specificity of milk ELISA test is high (99%); however, it lacks sensitivity during early stages of MAP infection [28]. This could perhaps have influenced the results of this study. Kirkpatrick et al. [8] reported that combining fecal culture and ELISA tests is more suitable in defining MAP infection status. Future studies could aim at determining EBVs for both tests combined to validate other JD SNPs. Two SNPs (rs41810662 and rs136182707) that were validated in this study were previously reported in a GWAS we conducted with Canadian Holstein cows for which no pedigree information was available [22]. Since no pedigree information was available for these Canadian Holsteins cows, their relatedness with the Canadian sires used in the current study could not be explained.

To the best of our knowledge, this is one of the first studies that considered validation of SNPs associated with JD reported in the literature using sire EBVs for milk ELISA test score. A follow-up study will consider search for gene variants in the same chromosomal areas as the identified SNP, which could play a role in JD resistance. One of the limitations associated with the current study is that validation was limited to a relatively small number of selected JD SNPs from GWAS and candidate gene studies from the literature. Future studies could consider validation of a larger number of reported JD SNPs using different (and ideally independent) dairy cattle populations.

The problems related with JD control, and the economic and welfare implications of the disease on the dairy industry, warrant extensive exploration of genetic selection as an alternative option to control JD. This begins with the validation of associated genetic markers. Validation of some of the SNPs in this study is a step in this direction and has prompted future consideration of similar validation studies using different phenotypes. It is a critical step before genetic marker-based selection can be implemented to breed for JD resistance in cattle.

## Conclusions

Using sire EBVs estimated for milk ELISA test scores, a total of four previously reported JD related SNPs were validated in this study which can be used to optimize genomic selection schemes. Future studies will consider exploring the genomic regions surrounding the validated SNPs for the presence of any candidate genes and genetic variants with relevance to JD.

## Methods

### Resource population, phenotype classification and genotyping

The EBVs for Holstein sires were calculated for milk ELISA test score as described earlier by Brito et al. [18]. These EBVs were estimated using univariate linear animal models using different data subsets in 3 different scenarios: a) the complete data set (all herds); b) herds with at least one suspect or test-positive animal (ELISA optical density ≥ 0.07); and c) SCEN3: herds with at least one test-positive animal (ELISA optical density ≥ 0.11). Out of 5285 sires with EBVs estimated for milk ELISA test score, a total of 498 were classified into high (*n* = 248) and low (*n* = 250) EBV groups for selective genotyping. The EBVs of the selected sires from both the groups ranged from − 0.0677 to 0.0275 with a standard deviation of 0.0151. All sires had at least 30 daughters. Genotyping was performed using a customized SNP panel comprised of 155 of the most prominent JD SNPs reported in the literature, which included SNP from studies that used phenotypes other than milk ELISA to define their case-control populations (such as serum ELISA, tissue and fecal MAP culture) and SNPs from candidate-gene studies [[9, 10, 12–14, 17, 22, 29–37]; Supplementary File A]. These SNPs were located on all *Bos taurus* autosomes (**BTA**) except BTA 24. A pedigree file containing 7479 animals was generated by tracing back the pedigrees of sires with data up to 18 generations ago.

### Quality control and statistical analyses

Quality control was applied to remove SNPs with minor allele frequency (**MAF**) less than 0.01 (or 1%). The association analysis was carried out using the Generalized Quasi-Likelihood Score method (**GQLS**) [38], implemented in the SNP1101 software [31]. The GQLS method is based on logistic regression and involves regression of EBVs versus SNP genotypes which are considered as response variables [38]. It further accounts for population substructure, by adjusting for relatedness among selected animals based on the pedigree-based relationship coefficients [39], and is not biased for selective genotyping. The GQLS statistical model can be defined as:

$$ {\mu}_i=E\ \left(\ {Y}_i|{\mathrm{X}}_i\right)=\frac{e^{\beta_0+{\beta}_1{\mathrm{X}}_i}}{1+{e}^{\beta_0+{\beta}_1{\mathrm{X}}_i}} $$

Where μ_i_ is the expected SNP allele frequency; **X**_**i**_ is the pseudo-phenotype (sire EBVs); **Y**_**i**_ is the genotype of the SNP, considering **Y**_**i**_ = 1/2 * (genotype code for the i^th^ animal). The genotypes were coded as “0”, “1”, and “2” based on the number of reference alleles, corresponding to the respective proportions of 0, 1/2 and 1; **β**_**0**_ is a constant and **β**_**1**_ is the slope coefficient. In order to verify the association between each marker and the trait (sire EBV for milk ELISA test score), the null hypothesis was: H_0_: β_1_ = 0, i.e. the marker is not associated with the trait; while the alternate hypothesis was: H_1_: β_1_ ≠ 0, i.e. the marker is associated with the trait. To account for testing of multiple comparisons and identify significant SNPs associated with sire EBVs, genome-wise false discovery rates (**FDR**) of 1 and 5% were applied. In addition to GQLS analysis, single-SNP regression analysis including the additive polygenic effect was carried out. Unlike GQLS analysis, single-SNP regression allows for accounting for polygenic effect. The single-SNP regression model can be defined as:

$$ \text{y} = \mu + b\text{x} + \text{Za} + \text{e} $$

where **y** is a vector with the sires’ EBV for milk ELISA test scores; **μ** is the overall mean value of the EBVs; b is the additive allele substitution effect (linear regression coefficient) of a SNP; **x** is the vector of number of copies of a given SNP allele (coded as 0, 1, or 2 for BB, AB, and AA, respectively); **Z** is the incidence matrix linking additive polygenic effects to bull EBVs; **a** is the vector of additive polygenic effects; and **e** is a vector of the residual effects. The model assumptions are as follows: **a** follows a normal distribution *N* (**0**, **Gσ**^**2**^_**a**_), in which **G** is the genomic relationship matrix [40], and σ^2^_a_ is the additive genetic variance.

## Supplementary information

**Additional file 1: Supplementary File A:** List of SNPs used in the custom genotype panel.

## Data Availability

The sire EBVs were provided by Brito et al. 2018 [18] and were stored in the Centre for Genetic Improvement of Livestock (CGIL) repository (http://cgil.uoguelph.ca/). These datasets are not publicly available due to on-going analyses but are available from the senior author (NK) on reasonable request.

## Abbreviations

JD: Johne’s disease
MAP: *Mycobacterium avium subsp. paratuberculosis*
SNPs: Single nucleotide polymorphisms
EBVs: Estimated breeding values
GQLS: General quasi likelihood scoring
MES: Marker-enhanced genetic selection
MAF: Minor allele frequency
